# Supplementary material for: Transcriptomic Insights into Mechanisms of Early Seed Maturation in the Garden Pea (Pisum sativum L.)
Source: Cells. 2020 Mar 23;9(3):779. doi: 10.3390/cells9030779 (PMC7140803; doi:10.3390/cells9030779)

Zhongwan−6(10) vs Zh

Global Summary

%DE = 0.87

genes with fdr < 0.2 = 1746 (1014 + / 732 −)

genes with fdr < 0.1 = 1644 (967 + / 677 −)

genes with fdr < 0.05 = 1493 (887 + / 606 −)

genes with fdr < 0.01 = 1235 (742 + / 493 −)

genes in genesets = 0

<FC> = 0

<t−score> = 4.49

<p−value> = 0

<fdr> = 0.13

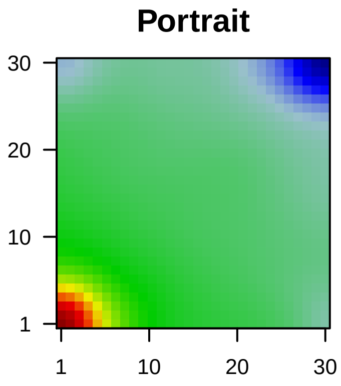

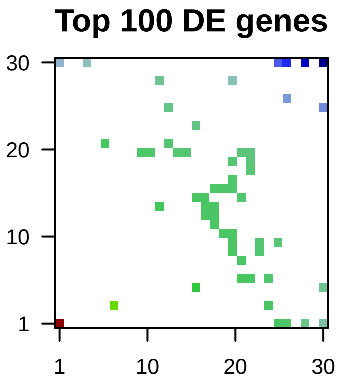

Global Genelist

Rank	ID	log(FC)	p−value	fdr	Metagene	Description
1	TRINITY_DN1	−0.06	7e−10	0.0000001	13 x 21	
2	TRINITY_DN8	0	1e−09	0.0000001	17 x 14	
3	TRINITY_DN8	0	1e−09	0.0000002	22 x 6	
4	TRINITY_DN7	0	3e−09	0.0000002	24 x 3	
5	TRINITY_DN3	0	3e−09	0.0000004	17 x 14	
6	TRINITY_DN1	−1.62	6e−09	0.0000004	30 x 30	
7	TRINITY_DN5	0	7e−09	0.0000004	17 x 14	
8	TRINITY_DN1	0	7e−09	0.0000004	28 x 1	
9	TRINITY_DN1	0	1e−08	0.0000004	18 x 13	
10	TRINITY_DN2	−2.38	1e−08	0.0000004	30 x 30	
11	TRINITY_DN7	0	1e−08	0.0000009	11 x 20	
12	TRINITY_DN1	0	2e−08	0.0000009	17 x 14	
13	TRINITY_DN1	−0.89	3e−08	0.0000009	25 x 30	
14	TRINITY_DN1	0	3e−08	0.0000009	18 x 16	
15	TRINITY_DN1	0	3e−08	0.0000009	18 x 14	
16	TRINITY_DN7	0	3e−08	0.0000009	21 x 6	
17	TRINITY_DN1	−0.02	3e−08	0.0000009	25 x 1	
18	TRINITY_DN7	0	3e−08	0.0000009	10 x 20	
19	TRINITY_DN1	0	4e−08	0.0000009	17 x 14	
20	TRINITY_DN1	0	4e−08	0.0000009	18 x 13	

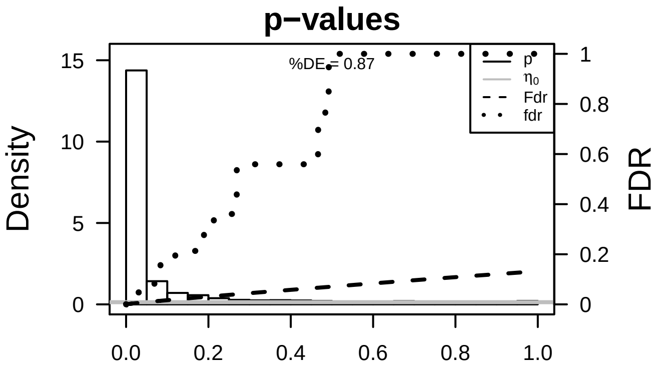

Supplement: Supplementary file 1 [file cells-09-00779-s001.zip › Supplementary materials/FileS4.zip!/FileS3/TFs - Results/Summary Sheets - Differences/Reports/Zhongwan.6.10..vs.Zhongwan.6.25..pdf]
